# Spontaneous Formation of Functional Structures in Messy Environments

**DOI:** 10.3390/life12050720

**Published:** 2022-05-11

**Authors:** Christian Mayer

**Affiliations:** Institute of Physical Chemistry, CENIDE, University of Duisburg-Essen, 45141 Essen, Germany; christian.mayer@uni-due.de; Tel.: +49-201-183-2570

**Keywords:** messy environments, order, complexity, function, selection, origin of life

## Abstract

Even though prebiotic chemistry initially deals with simple molecules, its composition rapidly gains complexity with oligomerization. Starting with, e.g., 20 monomers (such as the 20 proteinogenic amino acids), we expect 400 different dimers, 3,200,000 pentamers, or more than 10^13^ decamers. Hence, the starting conditions are very messy but also form a very powerful pool of potentially functional oligomers. A selecting structure (a “selector” such as membrane multilayers or vesicles) may pick and accumulate those molecules from the pool that fulfill a simple function (such as the suitability to integrate into a bilayer membrane). If this “selector” is, in turn, subject to a superimposed selection in a periodic process, the accumulated oligomers may be further trimmed to fulfill more complex functions, which improve the survival rate of the selectors. Successful oligomers will be passed from generation to generation and further improved in subsequent steps. After thousands of generations, the selector, together with its integrated oligomers, can form a functional unit of considerable order and complexity. The actual power of this process of random formation and selection has already been shown in laboratory experiments. In this concept paper, earlier results are summarized and brought into a new context.

## 1. The Creative Potential of Messy Prebiotic Chemistry

If we talk about a chemically messy environment, we usually have complex, chaotic systems in mind that contain a large variety of chemical compounds [1,2]. In principle, the full chemical space of small organic molecules, even with some restrictions in molecular size and contributing elements, contains millions of molecules [3,4]. Oligomers and polymers consisting of repetitive units with some limited variety form an interesting fraction of this chemical space [5]. This fraction is very likely to have special relevance for prebiotic chemistry for the simple reason that recent biological systems still make extensive use of it; proteins, nucleic acids, and carbohydrates are the most prominent examples. Other types of such polymers may have contributed in the past [6]. An interesting example is polyesters possibly formed by a variety of hydroxycarboxylic acids in analogy to proteins assembled from different amino acids [7,8]. In any case, we know that different types of polymers are actually the basis for the chemical variability of life. 

Therefore, in order to find a potential way out of prebiotic “messiness”, a good starting point is to think of a basic set of chain-forming molecules. If we choose the 20 proteinogenic amino acids for this purpose, we can expect 20 × 20 = 400 different dimers after the first condensation reaction. A further 4 condensation reactions lead to 20^5^ = 3.2 × 10^6^ pentamers, and 9 condensation reactions lead to more than 10^13^ different decamers. Hence, by oligomerization, the corresponding subset of the chemical space explodes into a huge variety. On the other hand, one has to consider the low concentrations of the oligomers in the thermal equilibrium. With knowledge of the average rate constants of condensation (k_c_) and hydrolysis (k_h_), these equilibrium concentrations c_n_ for oligomers with n repetitive units are determined by a set of differential equations [9]. For a situation with up to six units, the reactions that summarize all steps of formation and degradation for each oligomer A_n_—with A_1_ being the amino acid monomer—are listed in the following (water as a reaction partner is omitted in all cases for simplicity):
condensation reactions (with rate constant k_c_)hydrolysis reactions (with rate constant k_h_)A_1_  +  A_1_  ->   A_2_  x2 ^(1)^A_2_  ->   A_1_  +  A_1_A_1_  +  A_2_  ->   A_3_  x2 ^(1)^A_3_  ->   A_1_  +  A_2_  x2 ^(2)^A_1_  +  A_3_  ->   A_4_  x2 ^(1)^A_4_  ->   A_1_  +  A_3_  x2 ^(2)^A_1_  +  A_4_  ->   A_5_  x2 ^(1)^A_4_  ->   A_2_  +  A_2_A_1_  +  A_5_  ->   A_6_  x2 ^(1)^A_5_  ->   A_1_  +  A_4_  x2 ^(2)^A_2_  +  A_2_  ->   A_4_  x2 ^(1)^A_5_  ->   A_2_  +  A_3_  x2 ^(2)^A_2_  +  A_3_  ->   A_5_  x2 ^(1)^A_6_  ->   A_1_  +  A_5_  x2 ^(2)^A_2_  +  A_4_  ->   A_6_  x2 ^(1)^A_6_  ->   A_2_  +  A_4_  x2 ^(2)^A_3_  +  A_3_  ->   A_6_  x2 ^(1)^A_6_  ->   A_3_  +  A_3_

(1) These reactions have to be considered with a factor of two since each amino acid (or peptide) on the left side of the equation offers two possible reaction sites, -COOH and –NH_2_.
(2) These reactions have to be considered with a factor of two since the hydrolysis can occur at two different positions, each leading to an equivalent pair of products.

**Figure 1:**
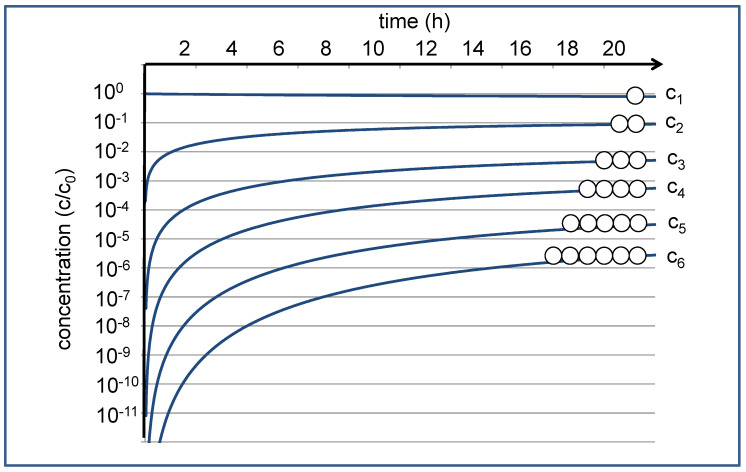
Time development of the relative concentrations of monomer (c_1_) and oligomers (c_2_–c_6_) according to the given reaction network, starting with a monomer solution at t = 0 [9]. The approximated time scale is dferived from NMR experiments at 120 °C [10], where k_c_ = 5.24 × 10^−3^ L/(mol∙min) and k_h_ = 5.76 × 10^−2^ L/min.

This reaction network leads to a set of differential equations, each characterized by averaged (second-order) rate constant k_c_ for the condensation reactions and a corresponding (pseudo-first-order) rate constant k_h_ for the hydrolysis reactions [9]. These coupled differential equations can be numerically solved, leading to a time development of the concentrations c_n,_ as shown in Figure 1. If we start with c_1_(t) = c_0_ at t = 0, the concentration c_1_(t) of the monomers hardly changes over time; the reduction by approximately 20% with respect to the starting concentration c_0_ is hardly visible on the logarithmic scale. The concentrations of the oligomers, on the other hand, vary dramatically. They start out at zero, then increase in the course of the condensation reactions, and finally approach the equilibrium values asymptotically over time. For the given settings of the rate constants, the equilibrium concentrations decrease by approximately an order of magnitude for each additional monomer unit. For the dimer, the equilibrium concentration c_2_ may still be in the range of 0.1 c_0_, which in practical experiments could be easily detected by NMR spectroscopy [10]. The equilibrium value for the hexamer, on the other hand, is expected near c_6_ = 3 × 10^−6^ c_0_, where in practice, it can only be observed by sensitive analytics.

Based on this estimation, one may be tempted to disregard the presence of the hexamers that only form about one-millionth of the mass of the total organic constituents in the given model system. However, this fraction has something like a powerful creative potential. It contains a theoretical set of 20^6^ = 64,000,000 different sequences. With ongoing condensation and hydrolysis processes, it forms a dynamic pool of potentially functional peptides. Even though their individual concentration may be astronomically small, they may be selected and accumulated out of the dynamic pool, while they will be constantly resupplied by the full set of equilibrium reactions summarized in Equations (1)–(6) in Ref. [9]. The situation could be seen as a representation of the infinite monkey theorem [11]; it is like a herd of monkeys repeatedly typing six consecutive letters representing the amino acid sequence of the hexamers. At this point, it just needs an efficient selector to pick out the meaningful or functional ones. Over time, the functional sequences will be typed again and again and just need to be collected.

Creative pools of this kind are especially active in high-temperature environments, such as near primordial volcanic islands [12], in hot springs [2,13,14], or tectonic fault zones [15,16,17]. The formation of chain molecules by condensation is significantly accelerated by periodic variation of the water activity. Such a repetitive switching between high and low water concentration can be induced by wet–dry cycling (as it would naturally occur at the shores of a hot pond in a daily rhythm) or by pressure variations connected to a phase transition of carbon dioxide resulting in a corresponding change in water solubility [16]. Caused by the periodic change of the water activity, the system remains in a constant non-equilibrium state, with longer oligomers being favored under dry conditions.

## 2. The Potential Nature of a “Selector”

Which system could have the power to actively select useful sequences out of this creative pool? In principle, any structure capable of molecular recognition and somehow complementary to this sequence would be suitable [2]. Selectively binding to an oligomer with a particular sequence, such a “selector” could form a stable complex that prevents hydrolysis of the oligomer and, therefore, efficiently removes it from the cycle of condensation and hydrolysis [13,14]. Consequently, the oligomer with this particular sequence would accumulate and reach concentrations far above the equilibrium values depicted in Figure 1. Of course, the question remains: where should the complementary sequence come from? It necessarily must have at least the same degree of complexity as the collected oligomer. Consequently, it would have to be formed by a separate process in advance.

Alternatively, and with significantly reduced selectivity, the growing oligomer could be encapsulated or bind to the surface of a mesoscopic structure formed by amphiphiles. Membrane structures such as multilayers, micelles, or vesicles could have this capability and act as efficient selectors for specific sequences of biopolymers such as polypeptides or nucleic acids [18,19,20,21,22,23,24,25].

**Figure 2:**
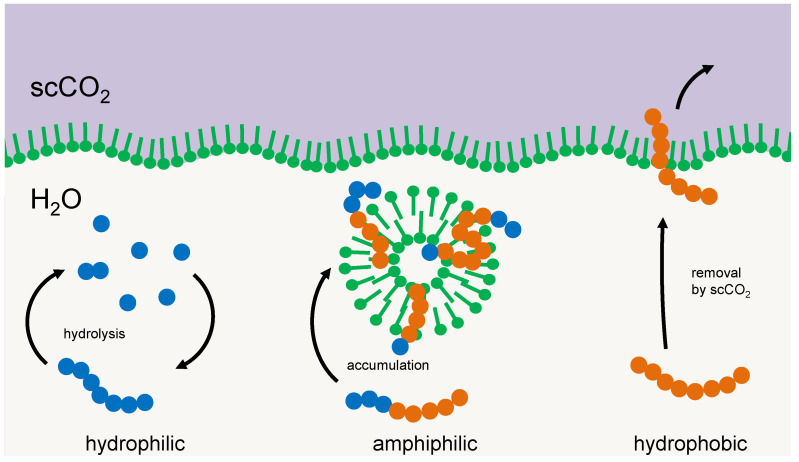
Example of a membrane structure acting as a selector for amphiphilic oligomers. Left: oligomers primarily made up of hydrophilic units (blue) form a cycle of condensation and hydrolysis in the aqueous phase. Right: oligomers made up primarily of hydrophobic units (red) tend to transfer into a hydrophobic environment, e.g., a separate phase of supercritical carbon dioxide. Center: amphiphilic oligomers integrate into membrane vesicles where they are protected against hydrolysis and phase transfer and accumulate over time.

Another principle for selection could be simple amphiphilic interactions between the selector and the particular oligomer. Membrane multilayers, micelles, or vesicles tend to integrate those oligomers that reflect the amphiphilicity profile of their main constituents [9,16,26]. With a suitable geometry of the hydrophilic and hydrophobic part of the oligomer chain, intermolecular interactions build-up and a negative enthalpy change occurs; hence the integration process would be energy driven. The dissipation of this energy would compensate for the loss in entropy that is connected to i) the integration process and ii) the selection of a particular fraction of the oligomers (the latter would correspond to the entropy of mixing that would occur if this fraction returns into the original pool). Integrated oligomers reside in a protected environment, being efficiently shielded against hydrolysis or elution. Consequently, they are being selected and accumulated, while the others either remain part of the cycle of hydrolysis and condensation or cross-phase boundaries [9]. An example of such a selector, membrane vesicles in a two-phase environment of supercritical CO_2_ and water in a high-pressure environment, is shown in Figure 2.

Membrane multilayers, micelles, and membrane vesicles are mesoscopic structures that form spontaneously whenever amphiphilic molecules occur at a suitable concentration [27,28]. Under hydrothermal conditions, the occurrence of those molecules is very likely. The simplest pathway to their prebiotic generation would be the formation of aliphatic chains with an oxidized initial methyl group by Fischer–Tropsch chemistry [29]. Molecules of this kind were detected in meteorites [30] as well as in recent tectonic fault systems [31] and in remnants of very old hydrothermal environments [17]. Therefore, one can consider membrane multilayers, micelles, or membrane vesicles as especially likely first selectors for prebiotic oligomers. This is even more so since all these structures are highly dynamic [27]. They easily form and disintegrate under cyclic conditions such as wet–dry cycling or periodic phase transitions [26], and they may have even undergone steps of self-organization on their own [32,33]. In addition, and this may be the most important aspect, they themselves can be subject to a second stage of selection.

## 3. The Selection of the Selector

**Figure 3:**
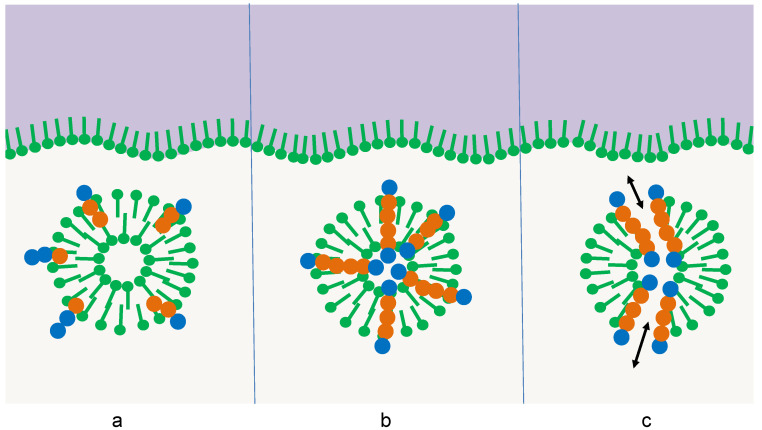
Three stages of the influence of the integrated oligomer on the survival of the membrane vesicle: (**a**) destabilizing effect, (**b**) stabilizing effect, (**c**) additional stabilizing function, e.g., the formation of pores leading to the relaxation of osmotic pressure [26].

Thus far, the accumulation of the oligomers would just follow one predetermined selection principle: the amphiphilicity profile. In this case, the selected oligomer would be of the same complexity as the selector itself, so no progress would be achieved. However, the selector itself may be subject to a superimposed selection process [9,26]. In the simplest case, its thermal stability could depend on the interaction with the integrated oligomer. This interaction may follow three different stages of complexity in its development (Figure 3):

(a) Oligomers with sequences of type (a) have a destabilizing effect on the membrane multilayer, the micelle, or the vesicle. In this case, the amphiphilic structure would come apart sooner than the competing ones, the amphiphiles would assemble at other surfaces, and the oligomers would be released. In the following, they are subject to hydrolysis just like the other oligomers in the pool and gain only a small temporary advantage.
(b) Oligomers with sequences of type (b) stabilize the membrane multilayer, the micelle, or the vesicle. The increase in thermal stability could derive from particular interactions between the oligomer and the adjacent amphiphiles. In this case, the lifetime of the structure would be extended, leading to extended protection of the oligomers with sequences of type (b) due to the reduced access of water molecules [26]. Hence, the sequences (b) would accumulate much more efficiently than the sequences (a).
(c) Oligomers with sequences of type (c) induce a more complex stabilizing function on the membrane multilayer, the micelle, or the vesicle. This effect goes beyond a simple thermodynamic stabilization by selective interactions. Instead, it compensates for destructive influences that shorten the lifetime of the amphiphilic structure. An example may be osmotic pressure, which regularly occurs during membrane formation. If an oligomer with a sequence (c) is capable of forming a transmembrane pore [26], this osmotic pressure can be released, leading to extended membrane longevity. Other specific functions may be the induction of a specific membrane curvature, the induction of a specific membrane mobility, or the accumulation of charges on the membrane surface. All these functions induced by sequences (c) could further extend the lifetime of the selector and hence give them an additional selective advantage.

The accumulation of the sequences (b) and (c) over time raises the chance that they will combine by condensation, leading to longer oligomers with superior survival strategies for the amphiphilic structures. After the stabilized selectors finally disintegrate, the oligomers of types (b) and (c) will be released into the pool, from where they will (at least partially) be collected by newly created selectors, creating a short-term molecular memory for the stabilized and functional structures. In all cases, the selection of the selectors leads to an increase in complexity far beyond the one that is observed for the basic selector itself and the oligomers that are initially accumulated.

**Figure 4:**
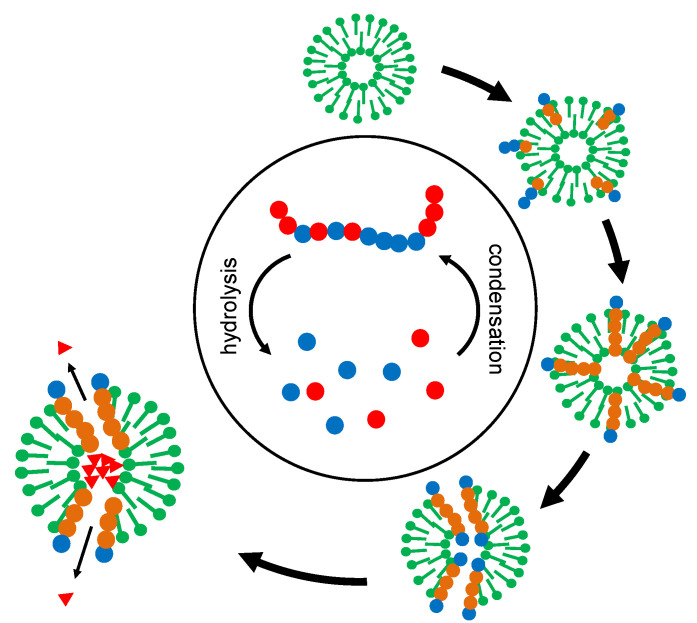
Superposition of two selection processes on the example of membrane vesicles. The vesicles (“selectors”) collect oligomers from a central pool of condensation and hydrolysis. At the same time, they themselves are being selected for stability. “Successful” selectors accumulate oligomers with stabilizing and functional properties. In the end, a function like the relaxation of a given concentration gradient (triangles) may even become a source of free energy, representing a starting point for energy metabolism.

The actual power of the overall development lies in the superposition of two selection processes (Figure 4). The original “creative pool” of oligomers (Figure 4, center) permanently delivers random sequences but also contains some chemical memory for successful stabilization and functionalization. The selector (in Figure 4, represented as membrane vesicles) picks out and integrates suitable oligomers and, by itself, is submitted to a selection process. In the course of this development, the selector develops more and more complex survival strategies.

In laboratory experiments, these conditions led to three kinds of effects caused by a particular selected oligomer with the sequence KSPFPFAA [26]:

(i) Thermal stabilization of the vesicle membrane;
(ii) An increase in the permeability of the vesicle membrane;
(iii) A decrease in the vesicle size.

**Figure 5:**
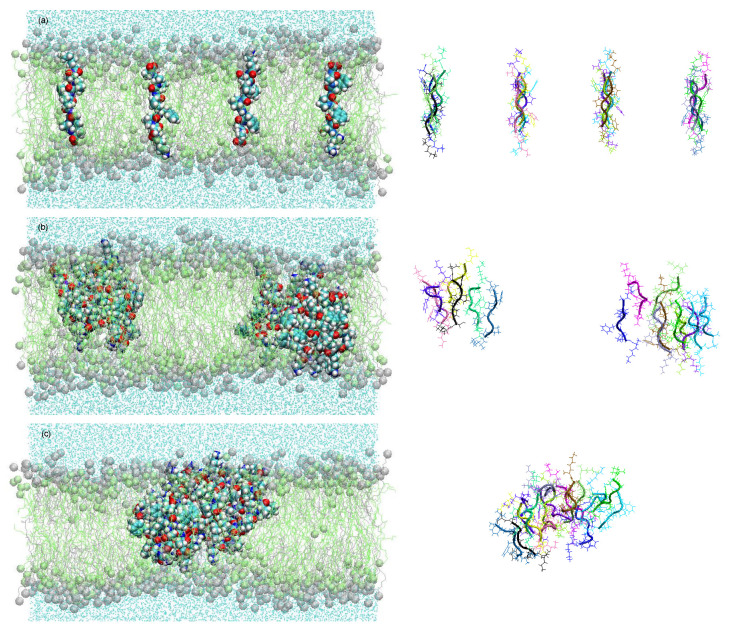
Membrane structure resulting from an experiment with superimposed selection processes in a molecular dynamics study [26,34]. Within 450 ns, the particularly successful oligomer KSPFPFAA adopts a stretched-out conformation across the membrane (**a**), starts to agglomerate in clusters (**b**), and forms pores with increased water permeability (**c**) [34].

All these effects can be interpreted as possible survival strategies for the vesicles. This is obvious in the case of the effect (i). In the case of (ii), the selection advantage may be given by the relaxation of the destructive osmotic pressure. In the case of (iii), the vesicles may reduce the risk of being mechanically destroyed by bubble formation and shear. A molecular dynamic simulation of the resulting membrane structure with the selected peptide KSPFPFAA is shown in Figure 5 [34]. Within a period of less than 0.5 µs, the oligomers form intra-membrane clusters with a hydrophilic pore in the center. This pore facilitates the passage of water molecules and, possibly, ions through the membrane, thereby reducing the osmotic pressure load.

## 4. Spontaneous Formation of Order and Complexity

The peptide cluster shown in Figure 5 is already of remarkable structural complexity. Nevertheless, it is obviously being formed in a spontaneous process from comparably simple molecules, all of them typical for a hydrothermal environment. It represents experimental evidence for the power of the described combination of random formation of oligomers with a two-step selection process. 

The thermodynamic driving force is basically the increase of the overall entropy, its rate *dS*/*dt* given by a balance equation [35]:

(1) $$\frac{dS}{dt}=\frac{d_{i}S}{dt}+\frac{d_{e}S}{dt}$$

where *d_i_S*/*dt* stands for the entropy increase of internal irreversible processes connected to periodic phase transitions together with all the chemical reactions they induce while the reaction mixtures inside the system are permanently kept in a non-equilibrium state. The term *d_e_S*/*dt* denotes the entropy flow rate due to the exchange of matter and energy with the environment, which is positive as well since processes like water evaporation or carbon dioxide flow are spontaneous on a large scale even if no internal processes occur.

In the proposed superimposed two-stage process, a tiny fraction of this overall entropy gain *dS*/*dt* is being sacrificed to generate local structural order by small-scale selection.

**Figure 6:**
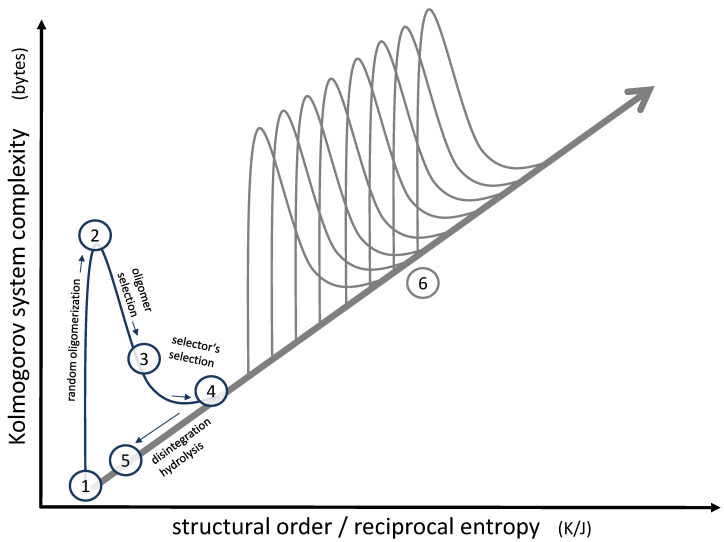
Representation of the effect of superimposed selection processes on a system’s order and complexity. A single generation of selectors follows positions (**1**) to (**5**). The effect of subsequent generations is shown at position (**6**). For a detailed description of positions (**1**) to (**6**), see text.

Focusing on this small local structure, the overall progress regarding complexity and order [36] may be illustrated, as shown in Figure 6. The molecules formed initially by hydrothermal chemistry (1) are low in complexity and occur in a diluted and mixed state, so they are low in structural order as well. As soon as those molecules form oligomers (2), the complexity rises dramatically (according to Kolmogorov’s definition of complexity, more bytes are needed for their description [37]), whereas the structural order remains more or less unchanged. This is basically the messy environment we are starting with. At this point, a primary selection process by a “selector” (3) reduces complexity but at the same time increases order (represented by a suitable sequence), the increase corresponding to the negative mixing entropy. The secondary selection process of the “selector” itself (4) follows the same pattern, leading to an even more significant increase in order due to the formation of distinct membrane structures, as illustrated in Figure 5. Eventually, even the most stable vesicles will disintegrate, thereby losing a large part of the original progress in complexity and order (5). However, some of the successful oligomers will remain in the pool; therefore, the following sequence will not have to start from scratch. Instead, the next step will begin at position (5) in the diagram. If the superimposed selection process is repeated in numerous subsequent generations, the overall development will follow the path (6), indicated on the right of Figure 6. Therefore, the endpoint of the superimposed selection will gradually shift along the diagonal in the diagram. In essence, it is this development of rising order and complexity along this diagonal which can lead to specific survival mechanisms and, eventually, to something like a primitive functional unit [36]. Peptides grown by the condensation of randomly formed short oligopeptides [38] may further support the early functionality, especially since they may have developed catalytic functions [39].

## 5. Conclusions

The described interaction between two superimposed selection processes has a considerable power to turn a “messy” environment (in a chemical sense) into a system that contains complex functional structures. During this process of random formation and selection, the system’s order and complexity increase gradually, eventually leading to structural functionality. The overall driving force is represented by the entropy increase of the vast majority of system components, which is only slightly reduced by the selected fractions of increased structural order. Overall, the superimposed selection process can lead to a significant degree of complexity and function of local structures.

## Data Availability

Not applicable.
